# *NAM* gene allelic composition and its relation to grain-filling duration and nitrogen utilisation efficiency of Australian wheat

**DOI:** 10.1371/journal.pone.0205448

**Published:** 2018-10-15

**Authors:** Zaid Alhabbar, Rongchang Yang, Angela Juhasz, Hu Xin, Maoyun She, Masood Anwar, Nigarin Sultana, Dean Diepeveen, Wujun Ma, Shahidul Islam

**Affiliations:** 1 Australia China Centre for Wheat Improvement, School of Veterinary and Life Sciences, Murdoch University, Murdoch, Western Australia, Australia; 2 Department of field crops, College of Agriculture and Forestry, Mosul University, Mosul, Iraq; 3 College of Plant Science and Technology, Huazhong Agricultural University, Wuhan, Hubei, China; 4 Department of Primary Industries and Regional Development, South Perth, Western Australia, Australia; 5 School of Veterinary and Life Sciences, Murdoch University, Murdoch, Western Australia, Australia; Institute of Genetics and Developmental Biology Chinese Academy of Sciences, CHINA

## Abstract

Optimising nitrogen fertiliser management in combination with using high nitrogen efficient wheat cultivars is the most effective strategy to maximise productivity in a cost-efficient manner. The present study was designed to investigate the associations between nitrogen utilisation efficiency (NUtE) and the allelic composition of the *NAM* genes in Australian wheat cultivars. As results, the non-functional *NAM-B1* allele was more responsive to the nitrogen levels and increased NUtE significantly, leading to a higher grain yield but reduced grain protein content. Nitrogen application at different developmental stages (mid-tillering, booting, and flowering) did not show significant differences in grain yield and protein content. The *NAM-A1* allelic variation is significantly associated with the length of the grain-filling period. While the *NAM-A1* allele *a* was associated with a short to moderate grain-filling phase, the alleles *c* and *d* were related to moderate to long grain-filling phase. Thus, selection of appropriate combinations of *NAM* gene alleles can fine-tune the duration of growth phases affecting sink-source relationships which offers an opportunity to develop high NUtE cultivars for target environments.

## 1. Introduction

Breeding cultivars with high nitrogen use efficiency (NUE) is essential for sustainable wheat production. Generally, NUE comprises two aspects, including nitrogen uptake efficiency (NUpE) that represents the capacity of plants to absorb nitrogen (N) from soil and nitrogen utilisation efficiency (NUtE) that represents the plant’s ability to use absorbed N to produce grain [1, 2]. Understanding the mechanisms regulating these two processes is essential to improve NUE in crop plants. The NUpE, and NUtE metabolic pathways are strongly influenced by genetic variation and environmental factors [3, 4]. Modern wheat cultivars have been generally selected under non-limiting N fertilisation levels in breeding programs, resulting in sub-optimal NUE [5, 6]. Unless NUE is improved in modern cultivars, the application of N fertilisers is expected to increase more than three-fold in the next 30 years to meet the increasing food demand [7, 8]. High N fertiliser usage means higher environmental pollution and production costs [9]. Thus, developing cultivars with high NUE becomes highly important in modern agriculture. High NUE cultivars can be described as being able to produce greater than average yields in low N environments [10]. They have also been defined as genotypes that can produce higher yields when additional N is provided. Timing and dosage of N addition is crucial when determining NUE. While additional N can usually increase grain yield, excess N has a deleterious effect on NUE [11, 12].

The *Gpc-B1* or *NAM-B1* gene commonly present in wild emmer wheats facilitates efficient translocation of nutrients to the grain [13] and is considered as a genetic factor influencing NUE. *Gpc-B1* has been reported to improve grain protein content (GPC) in bread wheat without reducing grain yield [14]. The identification and practical utilisation of *Gpc-B1* started when Avivi (1978) evaluated wild emmer wheat (*Triticum turgidum* ssp. *dicoccoides*) from Israel and found that it was associated with higher protein content and large grain size [15]. The functional *NAM-B1* allele, which encodes a transcription factor of the NAC family, accelerates senescence and increases nutrient remobilisation from leaf tissues into the developing grain [16]. *NAM-A1* is a gene with similar function to *NAM-B1*, with beneficial effects on grain nutritional quality and several bread-making properties [17]. However, the modern wheat cultivars that carry a non-functional *NAM-B1* allele result in delayed senescence and reduced wheat GPC by over 30% [18]. Leaf senescence can influence crop production in two ways, i.e. by modifying nutrient remobilisation efficiency or by affecting the duration of photosynthesis. Leaf senescence is a plant growth-dependent process that allows the transfer of nutrients from areas of lower nutrient requirement to areas of higher nutrient requirement driven by active cell development such as developing grains.

In contrast to the *NAM* gene studies, there are many reports arguing that stay-green cultivars with delayed senescence provide a longer grain-filling period through continued N uptake and translocation [19, 20]. These cultivars have greater N uptake, accumulation, and translocation capabilities which provide further metabolic gains in NUE [21]. In addition, delayed leaf senescence also provides further carbon and nitrogen to the plant roots during grain-filling, which increases the capacity to extract more N from the soil compared to shorter grain-filling cultivars [22–24]. In order to understand the impact of *NAM-1* gene alleles on the NUE and its components of wheats grown in environments with no limiting factors, we conducted the current study using a suite of Australian wheat cultivars that differ in *NAM-1* allelic compositions. Allelic effects on grain yield, protein content, and NUE components were studied based on various N application timing and dosage.

## 2. Materials and methods

### 2.1 Field trial design

A field trial was conducted at Broomehill, Western Australia consisting of 19 Australian wheat cultivars with different allelic combinations of *NAM-A1*, *B1* and *D1* genes (Table 1) [25]. Field trial was managed by the Department of Primary Industries and Rural Development (DPIRD) on a private crop field under a formal agreement between DPIRD and the owner. Soil nutrient composition was analysed before conducting the experiment (S1 Table) that showed the soil N content was lower than the usual which made the site N responsive. The Nitrogen treatment included three levels: 0 kg N ha^-1^, 50 kg N ha^-1^ and 100 kg N ha^-1^. The timing of the N application was synchronised to several Zadoks growth stages: T1 = 100% of N rate was applied at mid-tillering (Z22-Z24); T2 = 100% of N rate was applied at booting (Z43- Z45); and T3 = 50% of N rate was applied at mid-tillering, and 50% of N rate was applied at booting [26]. Flexi-N (42.2% of N) was applied as a source of N and includes three types of N: 50% urea, 25% nitrate, and 25% ammonium [27].

**Table 1:** Composition of *NAM-A1* and *NAM-B1* alleles of 19 Australian wheat cultivars.

Cultivars	*NAM-A1* allele	*NAM-B1* allele	*NAM-D1* allele	Maturity	GY Kg ha^-1^	GPC %	NUtE kg grain kg N^−1^	NUpE kg N kg N^−1^	NUE kg grain kg N^−1^
**Alsen**	c	Deletion	a	Mid	1467 e	15.90 a	3.20 e	9.50 c-f	30.70 gh
**Baxter**	a	Non-functional	a	Early—mid	1443 e	14.30 bc	3.70 d	9.20 d-f	35.30 e-g
**Bonnie-Rock**	a	Non-functional	a	Early	1989 cb	12.70 g-j	3.90 cd	11.70 ab	46.70 b-d
**Chara**	a	Non-functional	a	Early—mid	1520 de	13.60 de	3.80 cd	9.10 d-f	35.20 e-g
**Drysdale**	c	Mixed	a	Early	1152 f	13.20 d-g	3.20 e	8.50 e-g	26.30 h
**Excalibur**	b	Non-functional	a	Early	1960 cb	12.90 e-i	4.10 bc	11.20 a-c	49.20 bc
**Gladius**	c/d	Non-functional	a	Mid	1432 e	13.10 e-h	3.80 cd	8.80 e-g	34.20 fg
**Gregory**	c	Non-functional	a	Mid—long	1787 cd	12.40 h-k	4.00 cd	10.30 b-e	42.60 c-e
**H45**	a	Non-functional	a	Early	1628 de	11.90 k	4.40 ab	8.10 fg	36.00 e-g
**Kukri**	a	Non-functional	a	Early	1640 de	13.50 d-f	3.70 d	10.60 a-d	38.90 ef
**Livingston**	a	Non-functional	a	Early	1560 de	13.00 e-i	4.00 cd	8.70 e-g	34.40 fg
**Mace**	d	Non-functional	a	Early—mid	2295 a	11.90 k	4.60 a	12.20 a	56.40 a
**Pastor**	c	Non-functional	a	Early—mid	1594 de	12.60 g-j	3.70 d	9.60 c-f	38.20 e-g
**RAC875**	c	Non-functional	a	Mid—long	1755 cd	12.40 i-k	3.90 cd	9.80 c-f	38.10 e-g
**Spitfire**	a	Mixed	a	Early	1468 e	13.90 cd	3.80 cd	8.80 e-g	35.50 e-g
**Volcani**	c	Functional	a	Early	1064 f	14.60 b	3.30 e	7.30 g	24.30 h
**Westonia**	a	Deletion	a	Early—mid	2057 ab	12.20 jk	4.30 ab	11.30 a-c	50.80 ab
**Wyalkatchem**	a	Non-functional	a	Early—mid	2088 ab	12.90 f-j	4.30 ab	11.20 a-c	51.60 ab
**Yitpi**	d	Non-functional	a	Mid—long	1641 de	13.50 d-f	3.70 d	10.30 b-e	40.90 df

Grain yield (GY), grain protein content (GPC), N utilisation efficiency (NUtE), N uptake efficiency (NUpE), and N use efficiency (NUE). Within the columns in each factor, means followed by the same letter are not significantly different according to LSD (P = 0.05). *Note*: maturity data adapted from Bioplatforms Australia. Retrieved from https://data.bioplatforms.com/organization/about/bpa-wheat-cultivars

The plot size was 3 m × 1.25 m with a 0.5 m gap between plots. Sowing date was mid June, which is the recommended date for this part of Western Australian. Field trial was carried out as a split-plot design, with cultivars randomised as main plots, N treatment randomised as the subplots, and each treatment replicated three times.

### 2.2 Phenotyping and sample collection from field trial

Grain and straw samples were harvested when all plants were completely matured by visual inspection. Before mechanical harvesting, a quadrat of 0.44 m^2^ of plant material was cut off at ground level using a small hand harvester for yield component measurement. Grain and straw yield was estimated, and the grain protein content and residual N in straw were analysed using a FOSS XPS Near-infrared reflectance (NIR) equipment with a model 5000 spinning cup. NIR data analysis was collected using WinISI software (FOSS NIR Systems Inc., Laurel, MD, USA). The residual N concentration in straw was calculated using both free nitrogen and protein/amino-acid bound divided by 4.43 [28].

### 2.3 Glasshouse experiment design

Based on the field trial results, four cultivars: Westonia, Spitfire, Bethlehem, and Mace were selected for a glasshouse experiment. Three cultivars, Westonia, Spitfire, and Mace, showed contrasting NUE in the field trial and also comprise different *NAM-1* allelic compositions. One cultivar originated from Israel, namely Bethlehem was included in this study since it has the functional *NAM-B1* allele that is different from the other three cultivars. The unique characteristics of each cultivar were also been considered for cultivar selection: Mace (high-yielding), released in 2008 and rapidly became the dominant cultivar in Western Australian and accounted for 66.7% of the total area sown to wheat in 2016 [29]; Spitfire, known as a high protein content and N remobilisation cultivar; Westonia, high in both protein content and grain yield [30]; Bethlehem, high protein content and moderate grain yield [31].

Soil collected from the field trial was used in the glasshouse experiment. Plants were grown in a controlled temperature and light environment. The experiment was laid out in a complete randomised block design. The pot has a dimension of 190 mm height × 200 mm top diameter × 180 mm bottom diameter without holes to avoid leaching. The pots were watered manually. A base N dose of 20 N kg ha^-1^ was applied at sowing, coupled with P and K fertilisers. Three N rates 0, 50 and 100 kg N ha^-1^ were applied at mid-tillering, booting and flowering stages, as shown in Table 2. In order to achieve an adequate statistical power for data analysis and trait dissection, each treatment contains 12 replications in the glasshouse, making a total of 432 pots being planted.

**Table 2:** Nitrogen rates and timing of application in the glasshouse experiment.

		Tillering	Booting	Flowering
**0 kg** **N ha**^**-1**^		0%	0%	0%
**50 kg** **N ha**^**-1**^	T1	100%	0%	0%
	T2	0%	100%	0%
	T3	50%	50%	0%
	T4	40%	20%	40%
**100 kg** **N ha**^**-1**^	T1	100%	0%	0%
	T2	0%	100%	0%
	T3	50%	50%	0%
	T4	40%	20%	40%

### 2.4 Phenotyping and sample collection from glasshouse experiment

The anthesis time was estimated by the appearance of anthers on approximately 50% of all heads (Z61-Z65). Leaf tissue samples were collected at mid-tillering (Z22-Z24), booting (Z43- Z45), and flowering (Z65), just before the N application according to Zadoks’ scale of cereal growth stages [26]. The Leaves were frozen in liquid nitrogen and then stored at -80°C. The frozen leaves were ground to a fine powder in liquid nitrogen and used for RNA extraction and N content measurements of leaf tissue.

The vegetative phase duration was estimated as the period from sowing to flowering, and the grain-filling phase duration was estimated as the period from flowering to the physiological maturity [32, 33]. The grain, straw and root samples were harvested when all plants were considered completely mature by visual inspection. Plants were hand-harvested to measure yield components. The number of heads was counted in each plant. The heads were cut off and the remaining straw was cut at ground level. Roots were washed thoroughly with water using a 1 mm mesh sized sieve until totally free of soil. Roots of the two plants grown in the same pot were weighed together. The grain and straw yields were measured and the grain number was counted for each head. The TGW was measured by multiplying the weight of grains by 1000 divided by the number of grains per sample. The grain, straw and root samples were oven-dried separately in a forced air circulating dryer at 70°C for 72 hours. The total nitrogen content of straw and grains was analysed following the same procedure of field samples as detailed in the previous section. Harvest index (HI) and N harvest index (NHI) were obtained by calculating the ratio of grain or N at harvest to total above-ground biomass or N, respectively [34, 35]. N uptake efficiency (NUpE) was calculated as the ratio of aboveground N content to total N supply. N utilisation efficiency (NUtE) was calculated as the ratio of grain yield to aboveground N content. N use efficiency (NUE) was calculated as the ratio of grain yield to total N supply or multiplying NUpE by NUtE [4, 8].

### 2.5 Leaf N content analysis

Plant samples were dried at 40 °C, and 0.1 g leaf tissue was analysed for total nitrogen using the Dumas high-temperature combustion method (CSBP Laboratory, Western Australian). Samples were loaded into a combustion tube at 950 °C and flushed with oxygen. Gases generated from this process were measured using a thermal conductivity cell for nitrogen (according to the instruction manual).

### 2.6 Gene expression analysis

#### 2.6.1 Digital droplet PCR (ddPCR) for the cDNA standards in the qRT PCR

The gene copy number of total *NAM-1* genes (*NAM-A1*, *-B1* and *-D1)* in wheat cultivars was calculated based on the standards generated by a ddPCR. RNA was extracted from the flag leave of wheat cultivar Spitfire using a Qiagen RNeasy mini kit. The cDNA was then synthesised using the SensiFAST cDNA Synthesis Kit (BioLine, Alexandria, NSW, Australia). The ddPCR was conducted as described by Yang et. al. [36] with a slight modification using 1 μl cDNA as template instead of DNA. Forward primer 5’- TCA CTG CTC CAT CAT CAG GA, reverse primer 5’—GGC GTC GTC TGC TGT GAA C, and probe 6xFAM -5’ CAG CCA TTT CCT GGA GGG CCT were used in the ddPCR.

#### 2.6.2 qRTPCR for the quantification of *NAM* gene expression in four wheat cultivars at three growth stages

The RNA extraction and the synthesis of cDNA followed the protocols described above. The purified RNAs were quantified by NanoDrop ND-1000 spectrophotometer and their concentration adjusted to 50 ng/μl for qPCR, which was carried out in a Rotor-Gene Q (Qiagen, Hilden, Germany). The qPCR reaction contained 7.5 μl 2x qPCR master mix, 1 μl cDNA, 0.4 μl 10 pmol/μl forward and reverses primers, and 0.5 μl 10 pmol probe, as described above. A 1: 10 dilution dilustion series (5) of the standards generated from ddPCR was included in each run to generate the standard curves and calculate the number of *NAM* gene expressed copies.

### 2.7 Statistical analysis

Analysis of variance (ANOVA) was performed using the Genstat statistical software (Genstat Eighteenth Edition; 18.1.0.17005, 2015, UK) to determine genotype and nitrogen treatment effects at different times of application. In the case of significant differences based on ANOVA and F-values for treatment effects, LSD (p<0.05) test and standard deviation/error of means were used to identify significant means. Correlation analysis was conducted to investigate the relationship between the *NAM1* gene alleles and NUE components using Genstat.

## 3 Results

### 3.1 Influence of *NAM* genes under field conditions

#### 3.1.1 Grain yield, protein content, NUE, and its components were influenced by *NAM* gene allelic composition

A total of 19 wheat cultivars included in the field trial were compared to examine the influence of *NAM* gene allelic combinations on plant maturity type, grain yield, grain protein content, nitrogen use efficiency and its components (Table 1). Significant grain yield differences (P<0.001) were found among the 19 cultivars. Mace, Wyalkatchem, and Westonia produced the highest grain yield (Table 1). Results also indicated that the grain yield was significantly influenced by N rate (P<0.013). The maximum yield (1705 kg ha^-1^) was achieved at the highest level of N (100 kg N ha^-1^); while the lowest yield (1578 kg ha^-1^) was obtained from the control treatment (0 kg N ha^-1^, S2 Table). Similarly, significant differences (P<0.001) were observed for grain protein content among the cultivars. The grain protein content increased with the increase of N rate. Application of N at booting stage led to achieve maximum grain protein content (13.40% average). The highest grain protein content was obtained in cultivar Alsen, being 33.61% higher than that of H45 and Mace (Table 1). NUE and its components NUtE and NUpE were significantly influenced (P<0.001) by the cultivars and N rate. Cultivars Mace, Wyalkatchem, Westonia, Excalibur, and Bonnie-Rock attained the highest NUE, NUtE, and NUpE. The control treatment which only had the base N application (20 kg ha^-1^) produced the highest NUE, NUtE, and NUpE followed by 50 and 100 kg N ha^-1^.

#### 3.1.2 Correlation of *NAM-A1* and *-B1* alleles with phenotypes

Correlation analysis of *NAM-A1* and *-B1* alleles with phenotypes is presented in Table 3. A significant correlation was observed between *NAM-B1* allelic variation and yield, protein content, and NUtE. The functional *NAM-B1* allele produced a lower grain yield and NUtE but higher protein content. The *NAM-B1* deletion allele was positively correlated with grain protein content (Table 1). On the other hand, there was no significant correlation between *NAM-A1* allelic variation with yield or protein content alone. However, *NAM-A1* allelic variation was significantly correlated with plant maturity and NUtE (Table 3). *NAM-A1c* and *d* alleles were associated with low NUtE and longer maturity. It is worth pointing out that the *NAM-A1a* allele had both positive effects on grain yield and protein content even though such effect was not statistically significant based on the field trial data used in the current study.

**Table 3:** Correlations among allelic variation of *NAM-1* genes and agronomic traits of 19 wheat cultivars under field conditions.

	***NAM-A1***	***NAM-B1***	**Maturity**	**GPC**	**GY**	**NUpE**	**NUtE**	**NUE**
**NAM-A1**	1							
**NAM-B1**	-0.15	1						
**Maturity**	0.58***	0.37***	1					
**GPC**	0.07	-0.23**	0.04	1				
**GY**	-0.16	0.48***	0.06	-0.47***	1			
**NUpE**	-0.02	0.10	0.02	-0.38***	0.02	1		
**NUtE**	-0.29***	0.39***	-0.07	-0.78***	0.65***	0.44***	1	
**NUE**	-0.06	0.15	0.01	-0.47***	0.14	0.98***	0.56***	1

*, **, *** Significant at the 0.05, 0.01and 0.001 probability level, respectively. Grain protein content (GPC), Grain yield (GY), N uptake efficiency (NUpE), N utilisation efficiency (NUtE), and N use efficiency (NUE).

### 3.2 Influence of *NAM* genes under controlled environmental conditions

Four cultivars: Westonia, Spitfire, Bethlehem, and Mace had been selected for further investigation under controlled environment in glasshouse based on their contrasting responses to nutrient remobilisation in the field trial and *NAM* gene composition. As being investigated by this research group in another study [25], Bethlehem contain a functional *NAM-B1* gene, while Westonia has deletion and Mace has a non-functional allele. On the other hand Spitfire has been identified with the combination of both the functional and non-functional *NAM-B1* alleles. In the case of *NAM-A1* gene, Westonia and Spitfire contain allele *a*, while Bethlehem and Mace contain allele *d* (Table 4).

**Table 4:** Wheat cultivars carrying different *NAM-1* genes has various of vegetative, grain filling and total duration.

Cultivar	*NAM-A1* allele	*NAM-B1* allele	*NAM-D1* allele	Vegetative phase duration (days)	Grain-filling phase duration (days)	Days to maturity
**Spitfire**	a	Mixed	a	73	37	110
**Mace**	d	Non-functional	a	77	48	125
**Westonia**	a	Deletion	a	72	40	112
**Bethlehem**	d	Functional	a	69	53	122

#### 3.2.1 Grain yield and its components, aboveground biomass, HI and NHI

There were significant differences (P<0.001) in grain yield and yield components among the cultivars and N treatments. Interactions of N rate with both of the cultivars and time of application significantly influenced the grain yield and its components. The mean values for the cultivars across all N rates and times of application showed a 13.97% higher grain yield of Mace compared to Bethlehem and Spitfire (Table 5). Increasing N rates led to grain yield increases for all cultivars and 100 kg N ha^-1^ application produced the highest average yield (7.08 g plant^-1^). The interaction between cultivars and N rate had a highly significant impact on grain yield, demonstrated by the highest yield of Mace at 100 kg N ha^-1^ being 43.88% higher than that of Spitfire at 0 kg N ha^-1^ (S1 Fig). The N application time and rate were also interacting significantly, with the highest average grain yield (7.50 g plant^-1^) achieved from 100 kg N ha^-1^ applied at mid-tillering while the lowest average (6.20 g plant^-1^) obtained from the 0 kg N ha^-1^ in control treatment (S2 Fig). The highest number of grain head^-1^ was recorded from Mace (49.31 grain) while the lowest number was recorded from Spitfire (42.77 grain, Table 6). The time of N application also influenced the number of grain head^-1^. The highest number of grain head^-1^ (47.08 grain) was achieved by applying N at booting stage.

**Table 5:** Effects of four wheat cultivars, N application, and time of N application on NUE components.

Cultivars	AGB g plant^-1^	GY g plant^-1^	HI %	NHI %	NUtE g grain g N plant^-1^	NUpE g N plant^-1^ g N^-1^	NUE g grain g N^-1^
**Bethlehem**	11.31 b	6.37 b	0.56 b	0.89 a	7.17 b	17.95 b	128.70 b
**Mace**	12.27 a	7.26 a	0.59 a	0.89 a	7.66 a	19.21 a	147.15 a
**Spitfire**	11.46 b	6.39 b	0.56 b	0.87 b	6.88 b	18.79 a	129.28 b
**Westonia**	11.77 ab	6.63 b	0.56 b	0.87 b	7.05 b	19.03 a	134.16 b
**N Rates**							
**0**	10.95 c	6.20 c	0.57	0.88	7.42 a	36.95 a	274.17 a
**50**	11.77 b	6.71 b	0.57	0.88	7.13 b	11.86 b	84.56 b
**100**	12.39 a	7.08 a	0.57	0.89	7.02 b	7.43 c	52.16 c
**Time N app.**							
**T1**	11.91	6.70	0.56	0.88	7.18	18.73	134.48
**T2**	11.65	6.70	0.57	0.89	7.22	18.77	135.52
**T3**	11.76	6.70	0.57	0.88	7.23	18.75	135.56
**T4**	11.50	6.56	0.57	0.88	7.12	18.74	133.43

Aboveground biomass (AGB), grain yield (GY), harvest index (HI), N harvest index (NHI), N utilisation efficiency (NUtE), N uptake efficiency (NUpE), and N use efficiency (NUE). Within the columns in each factor, means followed by the same letter are not significantly different according to LSD (P = 0.05). Means with no letter are not statistically different (F > 0.05).

**Table 6:** Effects of four wheat cultivars, N application, and time of N application on agronomic traits.

Cultivars	Anthesis date Day	Grain number Seed head^-1^	Head number Head plant^-1^	TGW g	DRW g	GPC %	RNS %
**Bethlehem**	68.42 d	45.16 b	3.28 b	44.25 a	5.91 b	8.98 a	1.44 c
**Mace**	76.56 a	49.31 a	3.58 a	41.69 b	7.65 a	8.42 c	1.53 b
**Spitfire**	72.50 b	42.77 b	3.46 ab	41.76 b	6.07 b	8.73 b	1.61 a
**Westonia**	71.33 c	45.41 b	3.61 a	41.10 b	6.57 b	8.92 ab	1.52 b
**N Rates**							
**0**	72.75 a	44.83	3.18 c	42.40	6.21 b	8.51 c	1.47 b
**50**	71.85 b	46.99	3.50 b	42.09	5.96 b	8.77 b	1.55 a
**100**	72.00 b	45.17	3.77 a	42.11	7.49 a	9.01 a	1.56 a
**Time N app.**							
**T1**	72.53	44.83 ab	3.64	41.62 b	6.29	8.64	1.53
**T2**	72.08	47.08 a	3.45	41.37 b	6.36	8.79	1.53
**T3**	71.97	46.99 a	3.42	41.74 b	6.50	8.75	1.53
**T4**	72.22	43.75 b	3.42	44.06 a	7.05	8.86	1.52

Anthesis date, grain number, head number, thousand grain weight (TGW), dry root weight (DRW), grain protein content (GPC), residual N in straw (RNS). Within the columns in each factor, means followed by the same letter are not significantly different according to LSD (P = 0.05). Means with no letter are not statistically different (F > 0.05).

On the other hand, TGW was significantly influenced by cultivar and time of N application (p<0.001). However, there was no evidence that N rate had an influence on TGW. The maximum TGW (44.06 g) was achieved when N was applied at the latest stage (flowering). The result of interaction between cultivar and N rate showed that Bethlehem produced the highest TGW (45.96 g) at 0 kg N ha^-1^ treatment (Data not shown). HI and NHI were significantly influenced by cultivars and by the interaction of cultivars and N rates (p<0.001) where Mace produced the highest in both the cases. The aboveground biomass increased significantly with the increase of N rates (p<0.001).

Correlation analysis of *NAM-A1* and *-B1* alleles with phenotypes (Table 7) shows that the non-functional *NAM-B1* allele was positively correlated with both the grain yield and HI, while negatively correlated with TGW. The *NAM-A1a* cultivars showed an earlier senescence while *NAM-A1d* cultivar demonstrated a longer green period. The higher HI, NHI and TGW were achieved by the *NAM-A1d* allele compared to that of allele *NAM-A1a*, indicating a longer green period in glasshouse is desirable for achieving higher grain yield.

**Table 7:** Correlations among allelic variation of *NAM-1* genes and agronomic traits of four cultivars under glasshouse conditions.

	**NAM-A1**	**NAM-B1**	**DVet**	**DGF**	**DRW**	**TGW**	**GPC**	**GY**	**HI%**	**NHI%**	**NUpE**	**NUtE**
**NAM-A1**	1											
**NAM-B1**	0.01	1										
**DVet**	0.09	0.86***	1									
**DGF**	0.92***	-0.03	-0.20	1								
**DRW**	0.13	0.45**	0.29	0.12	1							
**TGW**	0.31*	-0.38*	-0.38*	0.22	0.01	1						
**GPC**	-0.12	-0.46**	-0.50**	0.00	-0.07	0.32	1					
**GY**	0.24	0.57***	0.48**	0.07	0.48**	-0.31	-0.48**	1				
**HI%**	0.39**	0.46**	0.47**	0.12	0.15	-0.15	-0.42*	0.65***	1			
**NHI%**	0.47***	0.16	-0.12	0.53***	0.07	0.40**	0.22	0.27	0.72***	1		
**NUpE**	-0.01	0.03	0.16	-0.08	-0.20	0.01	-0.46***	-0.52***	-0.06	-0.06	1	
**NUtE**	0.48***	0.35*	0.38**	0.34*	0.12	0.08	-0.68***	0.41**	0.81***	0.55***	0.37*	1

*, **, *** Significant at the 0.05, 0.01and 0.001 probability level, respectively. Duration of vegetative phase (DVet), grain-filling phase (DGF), dry root weight (DRW), thousand-grain weight (TGW), grain protein content (GPC), grain yield (GY), harvest index (HI), N uptake efficiency (NUpE), N utilisation efficiency (NUtE), and N use efficiency (NUE).

#### 3.2.2 Nitrogen content in leaf tissue

**Fig 1:**
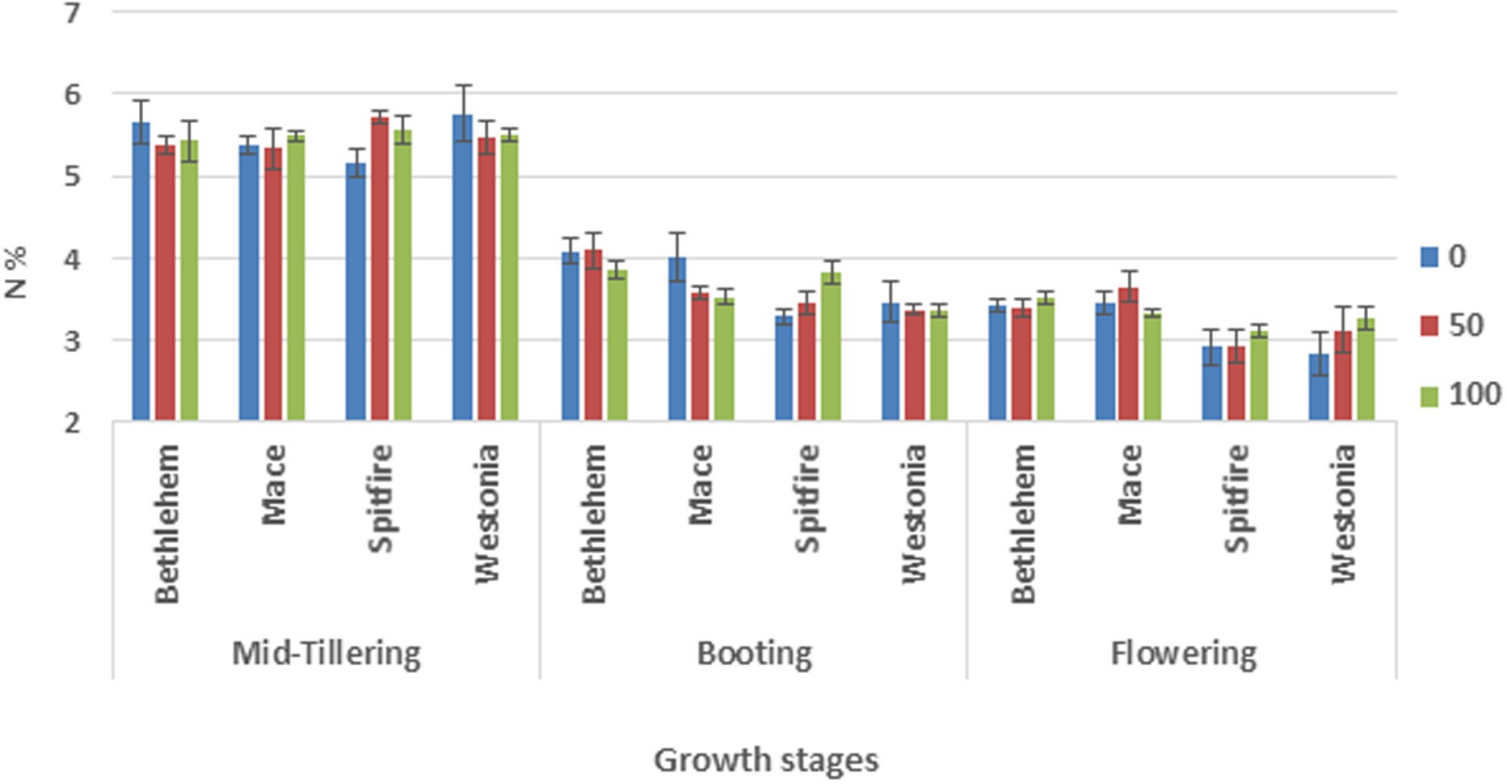
Effect of cultivars and N application on N content in leaf tissues at different growth stages.

Analysis of the data revealed significant effects of cultivar, N rate, and the time of N application on leaf N content. N content in leaves at three developmental stages (tillering, booting, and flowering) was significantly different (p<0.01). Fig 1 showed that all cultivars’ maximum N content in leaves was achieved at tillering stage (5.48%), while the lowest was at flowering stage (3.26%). Cultivar Bethlehem showed higher (p<0.01) N-content (4.30%) compared to Spitfire and Westonia (3.95 and 3.98%, respectively). The control treatment (0 kg N ha^-1^) recorded the highest (p<0.01) N content in leaf tissue compared to 50 and 100 kg N ha^-1^. The majority of the interactions between stages and other factors were significant for N content in leaf tissue.

#### 3.2.3 Grain protein content and residual N in straw

There was a significant difference (P<0.001) in the grain protein content and residual N in the straw between the cultivars and the N treatments. Cultivar Bethlehem produced the highest grain protein which was 7.98% more than the lowest (Mace), while it resulted the lowest residual N in the straw which was 6.15% less than the highest (Mace) (Table 6). Both grain protein content and residual N content in the straw increased when higher N rates were applied. Maximum grain protein content and residual N in the straw were obtained from 100 kg N ha^-1^, followed by 50 kg N ha^-1^, and the lowest from the control. The non-functional *NAM-B1* allele was negatively correlated with grain protein content (r = -0.46, P< 0.01). Allelic variation of *NAM-A1* did not show any significant correlation with grain protein content.

#### 3.2.4 Dry root weight (DRW)

DRW showed significant variations depending on the cultivars and N treatment (P<0.001). Cultivar Mace had the maximum DRW (7.65 g) while the lowest was with Bethlehem (5.91 g) (Table 6). DRW at 100 kg N ha^-1^ treatment was significantly higher than that of 0 and 50 kg N ha^-1^ (P<0.001). However, the effect of time of N application was not significant at P = 0.05. N applied at the latest stage (flowering) resulted in the highest DRW (7.05 g). DRW had a significant positive correlation with the non-functional *NAM-B1* allele (r = 0.45, P< 0.01).

#### 3.2.5 Nitrogen use efficiency (NUE) and its components

There were significant differences (P<0.001) in NUpE, NUtE, and NUE among the cultivars, N rate, and the interaction of cultivars with N rate. Cultivars Mace, Spitfire and Westonia exhibited significantly higher NUpE than Bethlehem. Similar to the field trial results, the control treatment (0 kg N ha^-1^) produced significantly higher NUpE than that of the 50 and 100 kg N ha^-1^ treatment. Mace at 0 kg N ha^-1^ had the highest NUpE (P<0.01). Mace also had the highest NUtE and overall NUE regardless of N application rate and timing of application (P<0.01). Mace at 0 kg N ha^-1^ had the highest NUE and NUtE (P<0.01). Among NUE and its components, only NUtE had a significant correlation with *NAM-A1* and *-B1* alleles (Table 7). NUtE was significantly correlated with the non-functional *NAM-B1* allele (r = 0.35, P <0.05), and the *NAM-A1*d allele (r = 0.48, P <0.001). Both the non-functional *NAM-B1* allele and *NAM-A1d* allele increased the NUtE.

### 3.3 *NAM-1* gene expression is influenced by cultivars and N treatments

**Fig 2:**
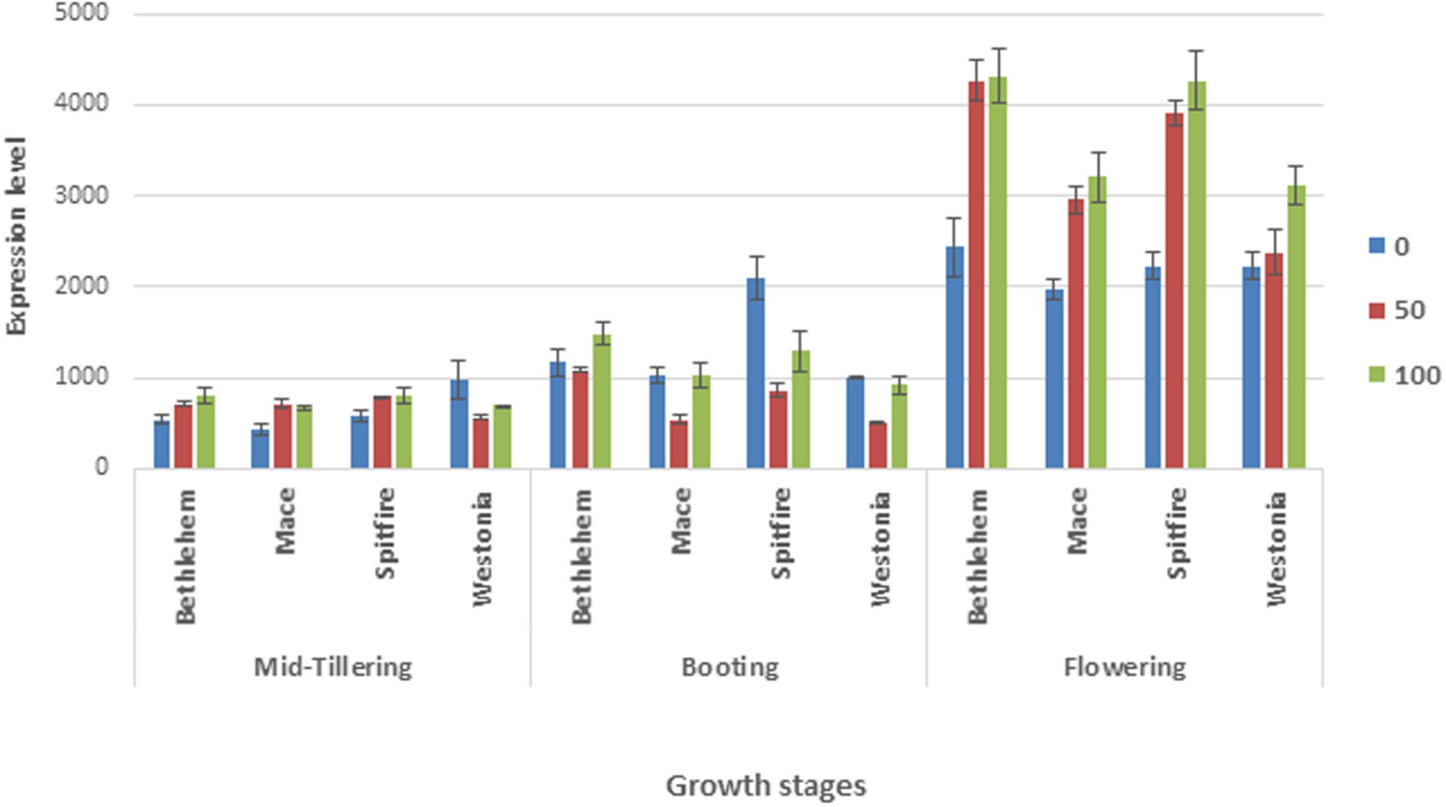
Efect of cultivars and N application on the total *NAM-1* gene expression at different growth stages.

Expression levels of total active *NAM-1* genes (*NAM-B1*, *-A1* and *-D1*) as determined by RT-PCR were significantly different (p<0.001) across cultivars, N rates, times of N application, and developmental stage (Fig 2). Averaged across cultivars, N rate, and time of N application, the highest total *NAM-1* gene expression level (3107) was occurred at flowering stage, followed by booting stage (1080), and mid-tillering stage (689, Fig 2). Averaged across growth stages, N rate, and time of application, Bethlehem (1868) and Spitfire (1864) exhibited higher total *NAM-1* gene expression levels than Mace (1393) and Westonia (1377). Gene expression level of the combined *NAM-1* genes increased (1389, 1608, and 1879) with the increase of N rates (0, 50 and 100) kg N ha^-1^, respectively. Averaged across cultivars, N rate, and growth stages, the highest combined *NAM-1* gene expression (1840) was observed when N was applied 50% at mid-tillering and 50% at booting stage, while the lowest *NAM-1* gene expression was observed when N was applied at once either at mid-tillering or at booting stage. *NAM-1* gene expression was significantly influenced by most interactions (S3 Fig).

## 4. Discussion

NUE is a complex trait that results from an interaction of several component traits such as grain and protein yield, NUtE, and NUpE. Recent studies have confirmed that around 60–95% of wheat grain N comes from the remobilisation of N stored in plant parts such as roots and shoots before anthesis (Kong et al. 2016, Barraclough et al. 2010, Hirel et al. 2007). The genetic factors involved in the absorption and utilisation of nutrients, such as differences in morphological, physiological and biochemical processes, have a large impact on NUE and its components. The *NAM-B1* transcription factor increases nutrient remobilisation and accelerates monocarpic senescence coupled with a slight yield penalty [16, 37], and is being considered as a genetic factor influencing NUE. *NAM-A1* is a gene with a similar function to *NAM-B1* involved in remobilising nutrients and accelerating senescence, with beneficial effects on protein content and yield [17]. However, *NAM-D1* has never been reported with any influence on NUE. This study was able to include only one cultivar in the field trail that harboring functional *NAM-B1*. This is because of the fact that functional *NAM-B1* is not common in bread wheat cultivars [13, 38, 39] which can be grown in Western Australian conditions. Only one Australian wheat cultivar has been identified having functional *NAM-B1* in the screening of 51 cultivars [25]. Thus to improve the strength of the correlation analysis we have included another cultivar (Bethlehem) from overseas (Israel) with functional *NAM-B1* in the glasshouse experiment.

In general, the non-functional *NAM-B1* allele showed a significant association with higher yield as evident by correlation analyses. For examples, Mace, Wyalkatchem, and Bonnie-Rock, which produced the highest grain yield, NUtE, NUpE, and NUE, carry a combination of non-functional *NAM-B1* and *NAM-A1a* or *d* alleles. However, independently from the presence of a functional gene or complete lack of *NAM-B1*, the presence of the *NAM-A1c* allele resulted in higher protein content, as seen in cultivars Volcani (functional *NAM-B1*, *NAM-A1c*), and Alsen and Drysdale (deletion *NAM-B1*, *NAM-A1c*).

The correlation analysis results showed a significant relationship between *NAM-1* allelic variation and maturity type. However, the interaction effect of *NAM-A1* and *NAM-B1* genes on determining the maturity of wheat cultivars also has been noticed. *NAM-A1* alleles *a* and *b* were exclusively characteristic of early and early to mid-maturing cultivars regardless of their *NAM-B1* allelic composition. On the other hand, *NAM-A1* alleles *c* and *d* were related to mid and late maturity only in combination with non-functional *NAM-B1* allele (Table 1), which is in concordance with the senescence-promoting role of the functional *NAM-B1* allele. In contrast, *NAM-A1* alleles *c* and *d* combined with functional/deletion *NAM-B1* alleles showed early and early to mid maturity in general. Based on the glasshouse results which represent wheat growing conditions without any limiting factors, we conclude, that the non-functional *NAM-B1* coupled with *NAM-A1* alleles *c* or *d* can be associated with high-yielding potential might be due to positive relation to the length of the grain-filling period. However, in most of the field conditions across the world with a range of complex environmental factors exist; different allelic effects have been detected. Under Western Australian conditions, our results have shown that the non-functional *NAM-B1* allele and the *NAM-A1a* alleles are favourable alleles for achieving higher grain yield and NUtE.

Based on the expression profiles shown at expVIPs (www.wheat-expression.com) [40], *NAM-B1* and *-A1* show the highest expression in the flag leaf and stamens, but is also expressed in the spikelet while *NAM-D1* is expressed in the spikelet and stamens during anthesis. The qRT-PCR analysis showed that the *NAM* gene expression reached the peak at flowering (Fig 2) in accordance with previously published articles confirming that the *NAM* gene function relates to senescence which ultimately influencing the maturity of the cultivars [13, 16–18, 41]. This was a total *NAM* gene expression which does not allow us to interpret the expression level of individual *NAM* gene or allele. However, the relative comparison indicated the total *NAM* gene expression was influenced by the types of alleles. For example, cultivars with functional *NAM-B1* allele (spitfire and Bethlehem) showed higher expression of total *NAM* gene than the cultivars with non-functional or deletion *NAM-B1* allele (Mace and Westonia). As presented in the Table 4 cultivar Spitfire and Westonia had the similar composition of *NAM-A1* and *D1* and also Mace and Bethlehem had the similar *NAM-A1* and *D1* composition. Thus the variation in the total gene expression between Spitfire and Westonia and also between Mace and Bethlehem is contributed by the variation at *NAM-B1* allele. On the other hand, comparison between the *NAM* gene expression of Mace and Westonia both of which carry similar type of *NAM-B1* allele showed that the variation of *NAM* allele doesn’t show much difference in total gene expression. It is worth mentioning that the less influence of *NAM-A1* allelic variation on the total *NAM* gene expression variation doesn’t disprove its function of determining maturity. This expression analysis also clearly demonstrated that *NAM-B1* functional allele boosted the gene expression when N fertilizer was added.

Although the four cultivars used in the glasshouse experiment represented early and early to mid-ripening types, we observed large differences in their development. The duration of the entire cycle was different in the four cultivars. The life cycle of Mace and Bethlehem was around two weeks longer than that of Spitfire and Westonia. Likewise, there was a difference between Mace and Bethlehem in the duration of vegetative and grain-filling periods. Based on these observations, we conclude that the four cultivars represent three different developmental mechanisms.

The first mechanism can be seen in Mace, which had a long vegetative phase (77 days) combined with a long grain-filling period (48 days). Accordingly, this cultivar had more chance to accumulate further N in the vegetative parts, which could then be remobilised to the grains during the grain-filling period. Mace, as a representative of high-yielding cultivars, carries a non-functional *NAM-B1* combined with the *NAM-A1d* allele (Table 4), which delayed the senescence and resulted in a low grain protein content. In contrast, this allelic combination produced higher grain yield, above-ground biomass, DRW and NUE and its components (Tables 5 and 6). Increasing the duration of the vegetative phase has the potential to improve the accumulation of total dry matter [42–44]. On the other hand, delayed leaf senescence can extend the duration of grain filling phase and thus enhance grain yield due to the addition in photosynthesis rate and grain filling capacity [45–47]. In the current study, the total dry matter and entire life cycle were significantly higher (P<0.01) in Mace resulted in higher grain yield, NUpE, NUtE, and NUE. Several studies have shown differences among cultivars in the pre-anthesis and post-anthesis build-up of grain yield and yield components [5, 48, 49]. The second mechanism can be observed in Bethlehem. This cultivar takes nearly the same number of days to reach at the end of the grain-filling stage as Mace. However, Bethlehem had a shorter vegetative phase (69 days) coupled with a long grain-filling period (53 days) to uptake and remobilise the N during the grain-filling period. The potential storage capacity of the grain is determined during the initial stage of endosperm cell division that is within the first 15 days after anthesis [22, 50]. The low grain yield and high grain protein content of Bethlehem, Alsen, Drysdale and Volcani can be explained by the different combinations of *NAM* gene alleles. Short vegetative phase related to the functional *NAM-B1* combined with low DRW, number of tillers (Heads plant^-1^), and number of grain head^-1^ (low total dry matter) resulted in decreased grain yield and yield components. However, a long grain-filling period is associated with the *NAM-A1d* allele, which resulted in an increased N uptake and remobilisation during the grain-filling period, led to an increased grain protein content and TGW. Our results are in consistent with the finding by Martre et al. (2007) ie., an increase in the duration of grain filling phases is a strategy to improve grain protein yield and NUE [22].

The third mechanism can be seen in Spitfire and Westonia, which represented similar growth types. Both cultivars have a medium vegetative phase (73 and 72 days respectively) followed by a very short grain-filling period (37 and 40 days, respectively). Medium vegetative phase was linked to absent or mixed *NAM-B1* allele and short grain-filling period related to *NAM-A1a* allele. Accelerated senescence might improve N remobilisation and grain protein content but it resulted in reduced TGW and lower grain number, thus resulting in decreased grain yield in cultivars carrying the *NAM-A1a* allele [17].

Differences between the three mechanisms can be explained in terms of the different combinations of *NAM-B1* and *-A1* alleles. The non-functional *NAM-B1* allele delayed leaf senescence [13, 39]. Increased duration of grain-filling due to the presence of *NAM-A1c* and *d* might provide more carbon and nitrogen, resulting a higher grain yield [17, 23]. At the same *NAM-A1* background, a negative correlation between the non-functional *NAM-B1* allele and grain protein content was observed. Remarkably, both functional *NAM-B1* and the deletion of *NAM-B1* gene correlate to the higher protein content.

The glasshouse result from the leaf tissue N analyses provides further support for the differences between the three mechanisms described above. In Fig 1, we can see that the maximum N accumulation in leaf tissues was at the early stage (mid-tillering) for all cultivars. At later stages (booting and flowering) the N was translocated to the developing grains. However, Spitfire and Westonia translocated the N earlier, because they have a shorter grain-filling period. The NHI data for the four cultivars provides further support regarding the negative impact of the short grain-filling period on grain yield. Spitfire and Westonia, which had a short grain-filling period, showed a low NHI compared with the other two cultivars (Table 5). NHI is an important parameter to measure the translocation efficiency of absorbed N from vegetative parts to the grain [20, 51]. To summarise, an increase in the duration of N accumulationat the leaves or the duration of N remobilisation to grains can both be good strategies to improve NUE.

Allele *NAM-A1a* is prevalent in early-ripening cultivars. The combinations of *NAM-A1c/d* with a functional *NAM-B1* gene result in higher protein content, as for example in Volcani and Bethlehem. However, it is important to measure gene expression levels, not only allelic presence or absence when determining the effect of *NAM-1* genes on yield, protein content, and NUtE.

## 5. Conclusions

Based on a large scale glasshouse experiment, three different developmental mechanisms involving the different duration of the grain-filling period were interpreted with the different combination of *NAM-1* genes. Presence of the non-functional *NAM-B1* allele is related to delayed leaf senescence meaning a longer grain-filling period and generally higher NUtE. On the other hand, functional *NAM-B1* allele and the *NAM-A1a* allele were associated with a shorter grain-filling period, making it useful in regions with a short rainfall season like Western Australia. A negative correlation between the non-functional *NAM-B1* allele and grain protein content was observed. In contrast, presence of functional *NAM-B1* allele or deletion of the *NAM-B1* gene are correlated with higher protein content. *NAM-A1* gene allelic composition was also strongly associated with maturity types. Cultivars with *NAM-A1a* and *b* alleles demonstrated early to mid maturity, while the cultivars with *NAM-A1c* and *d* alleles showed mid to late maturity at the same (non-functional) *NAM-B1* allele background.

The allelic effects are highly dependent on environmental conditions. The selection of specific combinations of *NAM-1* alleles offers an opportunity to develop new high NUtE cultivars for target environments. The higher N application had a positive effect on grain yield and its components as well as dry matter and dry root weight, while the timing and splitting of N applications had no obvious effect on NUE or its components. Late N application only increased grain protein content.

## Supporting information

S1 Table General characteristics of the soil used in the experiments. (DOCX)

S2 Table The effects of N rates and the time of N application on grain yield and protein content. (DOCX)

S1 Fig The interaction between the four cultivars and N rates on grain yield plant^-1^. (TIF)

S2 Fig The interaction between N rates and time of N application on grain yield plant^-1^. (TIF)

S3 Fig Effect of the time of N application on total *NAM* gene expression at different growth stage. (TIF)

## References

[1] Hirel B, Le Gouis J, Ney B, Gallais A. The challenge of improving nitrogen use efficiency in crop plants: towards a more central role for genetic variability and quantitative genetics within integrated approaches. Journal of Experimental Botany. 2007;58(9):2369–87. 10.1093/jxb/erm097 17556767

[2] Lea PJ, Azevedo RA. Nitrogen use efficiency. 1. Uptake of nitrogen from the soil. Annals of Applied Biology. 2006;149(3):243–7.

[3] Xu G, Fan X, Miller AJ. Plant nitrogen assimilation and use efficiency. Annual review of plant biology. 2012;63:153–82. 10.1146/annurev-arplant-042811-105532 22224450

[4] Moll R, Kamprath E, Jackson W. Analysis and interpretation of factors which contribute to efficiency of nitrogen utilization. Agronomy Journal. 1982;74(3):562–4.

[5] Guarda G, Padovan S, Delogu G. Grain yield, nitrogen-use efficiency and baking quality of old and modern Italian bread-wheat cultivars grown at different nitrogen levels. European Journal of Agronomy. 2004;21(2):181–92. 10.1016/j.eja.2003.08.001.

[6] Raun WR, Johnson GV. Improving nitrogen use efficiency for cereal production. Agronomy journal. 1999;91(3):357–63.

[7] Tilman D. Global environmental impacts of agricultural expansion: the need for sustainable and efficient practices. Proceedings of the National Academy of Sciences. 1999;96(11):5995–6000. 10.1073/pnas.96.11.5995 PMC34218 10339530

[8] Good AG, Shrawat AK, Muench DG. Can less yield more? Is reducing nutrient input into the environment compatible with maintaining crop production? Trends in plant science. 2004;9(12):597–605. 10.1016/j.tplants.2004.10.008 15564127

[9] Limon-Ortega A, Sayre KD, Francis CA. Wheat nitrogen use efficiency in a bed planting system in northwest Mexico. Agronomy Journal. 2000;92(2):303–8.

[10] Nyikako J, Schierholt A, Kessel B, Becker HC. Genetic variation in nitrogen uptake and utilization efficiency in a segregating DH population of winter oilseed rape. Euphytica. 2014;199(1–2):3–11.

[11] Macnack N, Khim BC, Mullock J, Raun W. In-Season Prediction of Nitrogen Use Efficiency and Grain Protein in Winter Wheat (*Triticum aestivum* L.). Communications in Soil Science and Plant Analysis. 2014;45(18):2480–94.

[12] Haile D, Nigussie D, Ayana A. Nitrogen use efficiency of bread wheat: Effects of nitrogen rate and time of application. Journal of soil science and plant nutrition. 2012;12(3):389–410.

[13] Asplund L, Bergkvist G, Leino MW, Westerbergh A, Weih M. Swedish spring wheat varieties with the rare high grain protein allele of *NAM-B1* differ in leaf senescence and grain mineral content. PloS one. 2013;8(3):e59704 10.1371/journal.pone.0059704 23555754 PMC3605437

[14] Eagles H, McLean R, Eastwood R, Appelbee M-J, Cane K, Martin P, et al High-yielding lines of wheat carrying *Gpc-B1* adapted to Mediterranean-type environments of the south and west of Australia. Crop and Pasture Science. 2014;65(9):854–61.

[15] Avivi L. High grain protein content in wild tetraploid wheat *Triticum dicoccoides* Korn. Proc 5th Int Wheat Genet Symp. 1978;1:372–80.

[16] Waters BM, Uauy C, Dubcovsky J, Grusak MA. Wheat (*Triticum aestivum*) *NAM* proteins regulate the translocation of iron, zinc, and nitrogen compounds from vegetative tissues to grain. Journal of experimental botany. 2009;60(15):4263–74. 10.1093/jxb/erp257 19858116

[17] Cormier F, Throude M, Ravel C, Gouis JL, Leveugle M, Lafarge S, et al Detection of *NAM-A1* natural variants in bread wheat reveals differences in haplotype distribution between a worldwide core collection and European elite germplasm. Agronomy. 2015;5(2):143–51.

[18] Uauy C, Brevis JC, Dubcovsky J. The high grain protein content gene *Gpc-B1* accelerates senescence and has pleiotropic effects on protein content in wheat. Journal of experimental botany. 2006;57(11):2785–94. 10.1093/jxb/erl047 16831844

[19] Barraclough PB, Howarth JR, Jones J, Lopez-Bellido R, Parmar S, Shepherd CE, et al Nitrogen efficiency of wheat: genotypic and environmental variation and prospects for improvement. European Journal of Agronomy. 2010;33(1):1–11.

[20] Hitz K, Clark AJ, Van Sanford DA. Identifying nitrogen-use efficient soft red winter wheat lines in high and low nitrogen environments. Field Crops Research. 2017;200:1–9.

[21] Christopher J, Manschadi A, Hammer G, Borrell A. Developmental and physiological traits associated with high yield and stay-green phenotype in wheat. Crop and Pasture Science. 2008;59(4):354–64.

[22] Martre P, Semenov MA, Jamieson PD. Simulation analysis of physiological traits to improve yield, nitrogen use efficiency and grain protein concentration in wheat. Frontis. 2007;21:179–99.

[23] Borrell AK, Hammer GL, Oosterom EV. Stay—green: A consequence of the balance between supply and demand for nitrogen during grain filling? Annals of Applied Biology. 2001;138(1):91–5.

[24] Richards R. Selectable traits to increase crop photosynthesis and yield of grain crops. Journal of experimental botany. 2000;51(suppl_1):447–58. 10938853 10.1093/jexbot/51.suppl_1.447

[25] Yang R, Juhasz A, Zhang Y, Chen X, Zhang Y, She M, et al. Molecular characterization of the NAM-1 genes in Australia wheat varieties Crop and Pasture Science: submitted. 2018.

[26] Zadoks JC, Chang TT, Konzak CF. A decimal code for the growth stages of cereals. Weed research. 1974;14(6):415–21.

[27] CSBP. The Flexi-N Range is a locally developed liquid fertilisers which apply nitrogen, sulphur and potassium evenly and accurately. 2012. https://csbp-fertilisers.com.au/fertiliser-products/liquid-fertilisers/flexi-n-range.

[28] Yeoh H-H, Wee Y-C. Leaf protein contents and nitrogen-to-protein conversion factors for 90 plant species. Food Chemistry. 1994;49(3):245–50.

[29] Department of Agriculture and Food WA, DAFWA. Area sown of wheat varieties in Western Australia 2008–2015 2016. https://www.agric.wa.gov.au/wheat/area-sown-wheat-varieties-western-australia-2008-2015.

[30] Balotf S, Islam S, Kavoosi G, Kholdebarin B, Juhasz A, Ma W. How exogenous nitric oxide regulates nitrogen assimilation in wheat seedlings under different nitrogen sources and levels. PloS one. 2018;13(1):e0190269 10.1371/journal.pone.0190269 29320529 PMC5761883

[31] Millet E, Rong J-K, Qualset C, McGuire P, Bernard M, Sourdille P, et al Production of chromosome-arm substitution lines of wild emmer in common wheat. Euphytica. 2013;190(1):1–17.

[32] Tao F, Zhang S, Zhang Z. Spatiotemporal changes of wheat phenology in China under the effects of temperature, day length and cultivar thermal characteristics. European Journal of Agronomy. 2012;43:201–12.

[33] Phillip B, Jan E, Nathan F, Tim M, Bill M, Karen R, et al. Wheat growth & development 2008. https://www.dpi.nsw.gov.au/.

[34] Gaju O, Allard V, Martre P, Snape J, Heumez E, Le Gouis J, et al Identification of traits to improve the nitrogen-use efficiency of wheat genotypes. Field Crops Research. 2011;123(2):139–52. 10.1016/j.fcr.2011.05.010.

[35] Siddique K, Kirby E, Perry M. Ear: stem ratio in old and modern wheat varieties; relationship with improvement in number of grains per ear and yield. Field Crops Research. 1989;21(1):59–78.

[36] Yang R, Paparini A, Monis P, Ryan U. Comparison of next-generation droplet digital PCR (ddPCR) with quantitative PCR (qPCR) for enumeration of Cryptosporidium oocysts in faecal samples. International journal for parasitology. 2014;44(14):1105–13. 10.1016/j.ijpara.2014.08.004 25229177

[37] Borrill PG. The NAM-B1 transcription factor and the control of grain composition in wheat: Doctoral dissertation, University of East Anglia; 2014.

[38] Masclaux-Daubresse C, Reisdorf-Cren M, Orsel M. Leaf nitrogen remobilisation for plant development and grain filling. Plant Biology. 2008;10(s1):23–36. 18721309 10.1111/j.1438-8677.2008.00097.x

[39] Hagenblad J, Asplund L, Balfourier F, Ravel C, Leino MW. Strong presence of the high grain protein content allele of *NAM-B1* in Fennoscandian wheat. Theoretical and Applied Genetics. 2012;125(8):1677–86. 10.1007/s00122-012-1943-2 22850788

[40] Borrill P, Ramirez-Gonzalez R, Uauy C. expVIP: a customisable RNA-seq data analysis and visualisation platform opens up gene expression analysis. Plant physiology. 2016:pp. 01667.2015. 10.1104/pp.15.01667 PMC4825114 26869702

[41] Uauy C, Distelfeld A, Fahima T, Blechl A, Dubcovsky J. A NAC gene regulating senescence improves grain protein, zinc, and iron content in wheat. Science. 2006;314(5803):1298–301. 10.1126/science.1133649 17124321 PMC4737439

[42] Fischer RA. The effect of duration of the vegetative phase in irrigated semi-dwarf spring wheat on phenology, growth and potential yield across sowing dates at low latitude. Field Crops Research. 2016;198:188–99. 10.1016/j.fcr.2016.06.019.

[43] Gebeyehou G, Knott D, Baker R. Relationships among Durations of Vegetative and Grain Filling Phases, Yield Components, and Grain Yield in Durum Wheat Cultivars 1. Crop Science. 1982;22(2):287–90.

[44] Alagarswamy G, Bidinger FR. The influence of extended vegetative development and d2 dwarfing gene in increasing grain number per panicle and grain yield in pearl millet. Field Crops Research. 1985;11:265–79. 10.1016/0378-4290(85)90108-X.

[45] Borrill P, Fahy B, Smith AM, Uauy C. Wheat grain filling is limited by grain filling capacity rather than the duration of flag leaf photosynthesis: a case study using *NAM* RNAi plants. PloS one. 2015;10(8):e0134947 10.1371/journal.pone.0134947 26241955 PMC4524614

[46] Hawkesford MJ, Araus JL, Park R, Calderini D, Miralles D, Shen T, et al Prospects of doubling global wheat yields. Food and Energy Security. 2013;2(1):34–48.

[47] Gebeyehou G, Knott D, Baker R. Rate and Duration of Grain Filling in Durum Wheat Cultivars 1. Crop Science. 1982;22(2):337–40.

[48] Arduini I, Masoni A, Ercoli L, Mariotti M. Grain yield, and dry matter and nitrogen accumulation and remobilization in durum wheat as affected by variety and seeding rate. European Journal of Agronomy. 2006;25(4):309–18. 10.1016/j.eja.2006.06.009.

[49] Przulj N, Momcilovic V. Genetic variation for dry matter and nitrogen accumulation and translocation in two-rowed spring barley: II. Nitrogen translocation. European Journal of Agronomy. 2001;15(4):255–65. 10.1016/S1161-0301(01)00108-3.

[50] Jenner C, Ugalde T, Aspinall D. The physiology of starch and protein deposition in the endosperm of wheat. Functional Plant Biology. 1991;18(3):211–26.

[51] Fageria N. Nitrogen harvest index and its association with crop yields Journal of Plant Nutrition. 2014;37(6):795–810.

