# Supplementary material for: NAM gene allelic composition and its relation to grain-filling duration and nitrogen utilisation efficiency of Australian wheat
Source: PLoS One. 2018 Oct 15;13(10):e0205448. doi: 10.1371/journal.pone.0205448 (PMC6188794; doi:10.1371/journal.pone.0205448)
Supplement: S1 Table — (DOCX) [file pone.0205448.s001.docx]

S1 Table General characteristics of the soil used in the experiments

| Sample spot | Lat | Long | pH [1:5 soil/CaCl2] | pH [1:5 soil/water] | EC [1:5] (dS/m) | Organic C [W&B] (%) | NO3-N [KCl] (mg/kg) | NH4-N [KCl] (mg/kg) | P [Colwell] (mg/kg) | PBI+ColP | K [Colwell] (mg/kg) | S [KCl-40] (mg/kg) | Texture | Colour | Gravel (%) | Sample depth cm |
| --- | --- | --- | --- | --- | --- | --- | --- | --- | --- | --- | --- | --- | --- | --- | --- | --- |
| 1 | -33.86308 | 117.76316 | 5.30 | 5.90 | 0.04 | 1.58 | 24 | 9 | 25 | 31 | 45 | 9 | Loamy Sand | Light Grey | 5 | 0-10 |
| 2 | -33.86287 | 117.76300 | 5.10 | 5.70 | 0.05 | 1.64 | 32 | 5 | 16 | 26 | 41 | 8 | Loamy Sand | Light Grey | 5 | 0-10 |
| 3 | -33.86270 | 117.76272 | 5.60 | 6.20 | 0.11 | 2.00 | 19 | 6 | 24 | 28 | 111 | 9 | Loamy Sand | Light Grey | 5 | 0-10 |
| 4 | -33.70309 | 117.42862 | 4.60 | 5.20 | 0.14 | 1.31 | 33 | 5 | 40 | 21 | 54 | 16 | Loamy Sand | Light Grey | 5 | 0-10 |
| 5 | -33.86246 | 117.76297 | 5.50 | 6.20 | 0.03 | 1.80 | 18 | 7 | 22 | 17 | 76 | 6 | Loamy Sand | Light Grey | 5 | 0-10 |
| 6 | -33.86199 | 117.76311 | 5.10 | 5.90 | 0.05 | 1.78 | 25 | 4 | 27 | 24 | 69 | 10 | Loamy Sand | Light Grey | 5 | 0-10 |
| 7 | -33.86216 | 117.76264 | 5.20 | 6.00 | 0.04 | 1.87 | 21 | 4 | 23 | 21 | 52 | 6 | Loamy Sand | Light Grey | 5 | 0-10 |
