# Supplementary material for: NAM gene allelic composition and its relation to grain-filling duration and nitrogen utilisation efficiency of Australian wheat
Source: PLoS One. 2018 Oct 15;13(10):e0205448. doi: 10.1371/journal.pone.0205448 (PMC6188794; doi:10.1371/journal.pone.0205448)
Supplement: S2 Table — (DOCX) [file pone.0205448.s002.docx]

S2 Table. The effects of N rates and the time of N application on grain yield and protein content

| **N application** | | **GY**  Kg ha^-1^ | **GPC**  % |
| --- | --- | --- | --- |
| **N rates** | **0** | 1578 b | 12.70 b |
|  | **50** | 1698 a | 13.30 a |
|  | **100** | 1705 a | 13.50 a |
| **Time of N application** | **T1** | 1678 | 13.00 b |
|  | **T2** | 1653 | 13.10 b |
|  | **T3** | 1648 | 13.40 a |

Grain yield (GY) and grain protein content (GPC). Within the columns in each factor, means followed by the same letter are not significantly different according to LSD (P = 0.05).
