# Supplementary figures and images for: NAM gene allelic composition and its relation to grain-filling duration and nitrogen utilisation efficiency of Australian wheat
Source: PLoS One. 2018 Oct 15;13(10):e0205448. doi: 10.1371/journal.pone.0205448 (PMC6188794; doi:10.1371/journal.pone.0205448)

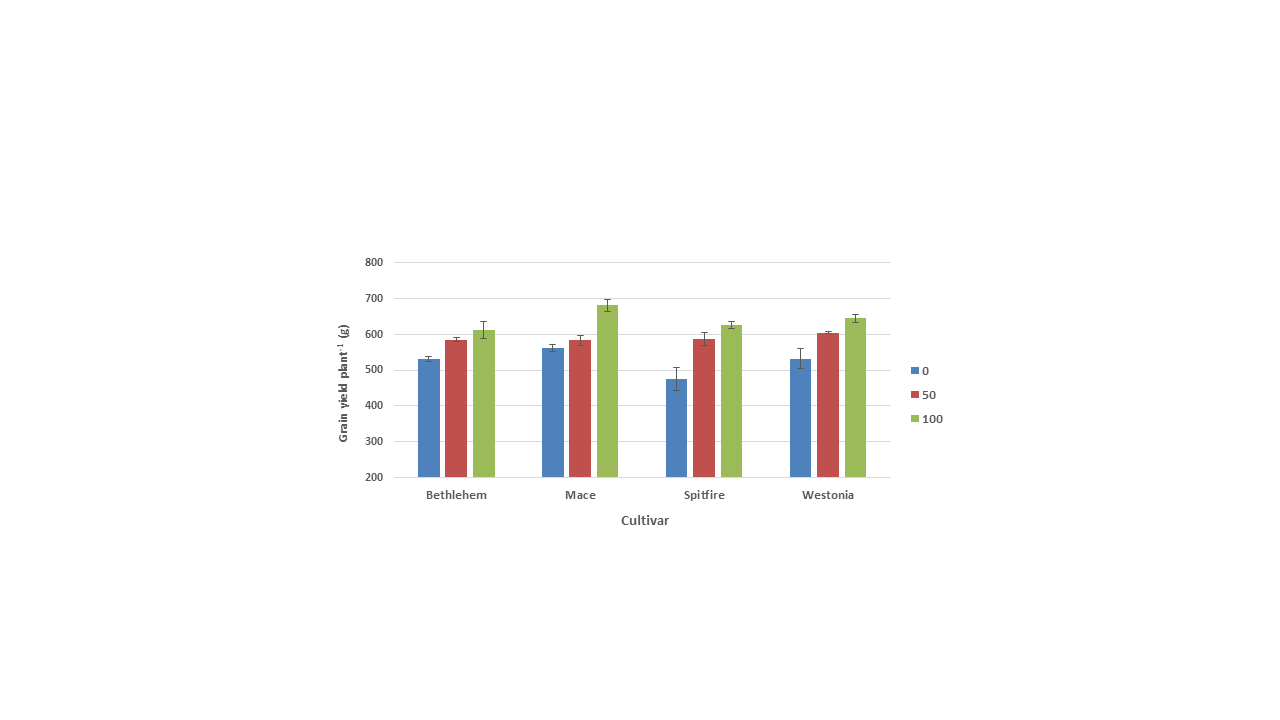

Supplement: S1 Fig — (TIF) [file pone.0205448.s003.tif]

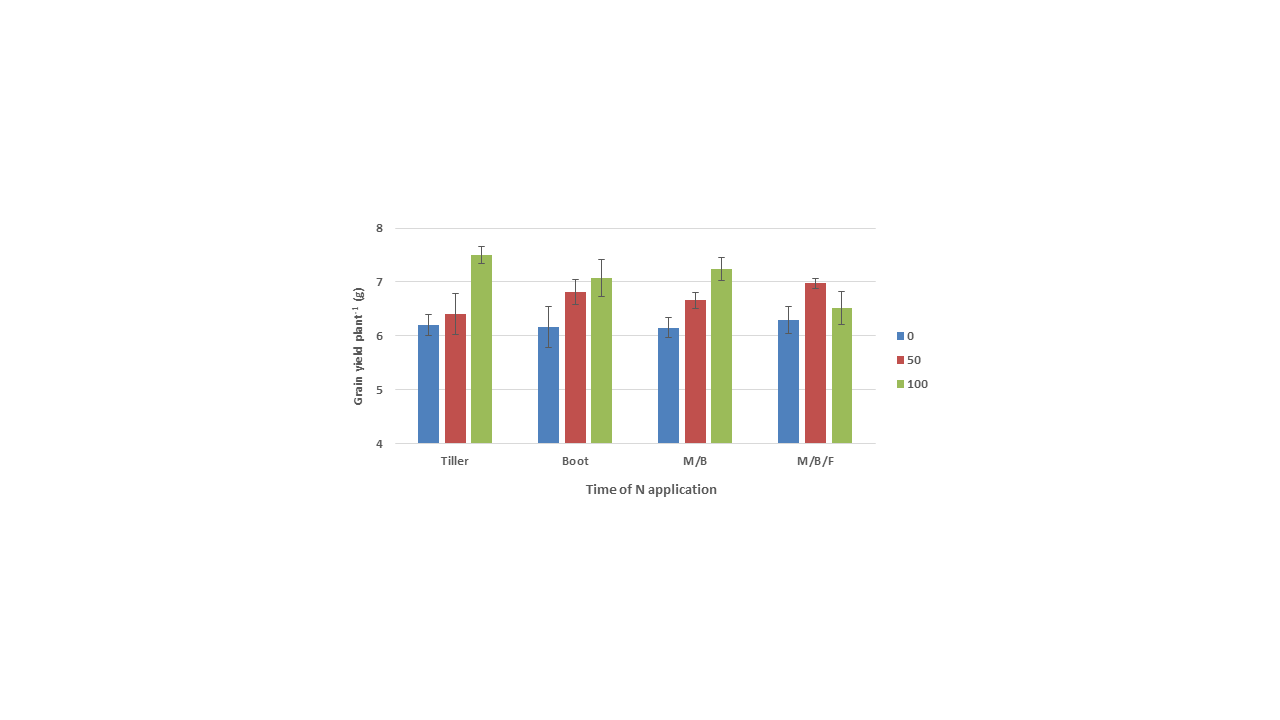

Supplement: S2 Fig — (TIF) [file pone.0205448.s004.tif]

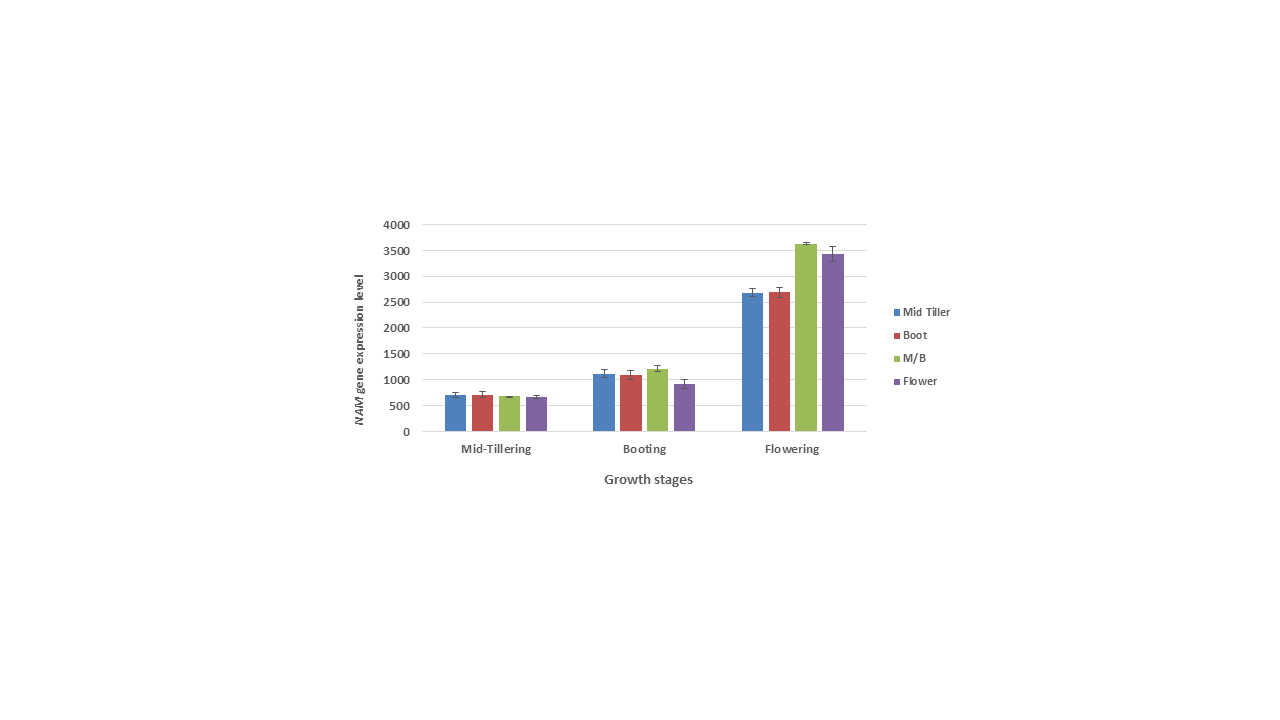

Supplement: S3 Fig — (TIF) [file pone.0205448.s005.tif]
